# Mechanical stability of bivalent transition metal complexes analyzed by single-molecule force spectroscopy

**DOI:** 10.3762/bjoc.11.91

**Published:** 2015-05-15

**Authors:** Manuel Gensler, Christian Eidamshaus, Maurice Taszarek, Hans-Ulrich Reissig, Jürgen P Rabe

**Affiliations:** 1 Department of Physics & IRIS Adlershof, Humboldt-Universität zu Berlin, Newtonstr. 15, D-12489 Berlin, Germany; 2 Institut für Chemie und Biochemie, Freie Universität Berlin, Takustr. 3, D-14195 Berlin, Germany

**Keywords:** molecular rupture mechanism, multivalency, malleability, pyridine coordination compounds, scanning force microscopy

## Abstract

Multivalent biomolecular interactions allow for a balanced interplay of mechanical stability and malleability, and nature makes widely use of it. For instance, systems of similar thermal stability may have very different rupture forces. Thus it is of paramount interest to study and understand the mechanical properties of multivalent systems through well-characterized model systems. We analyzed the rupture behavior of three different bivalent pyridine coordination complexes with Cu^2+^ in aqueous environment by single-molecule force spectroscopy. Those complexes share the same supramolecular interaction leading to similar thermal off-rates in the range of 0.09 and 0.36 s^−1^, compared to 1.7 s^−1^ for the monovalent complex. On the other hand, the backbones exhibit different flexibility, and we determined a broad range of rupture lengths between 0.3 and 1.1 nm, with higher most-probable rupture forces for the stiffer backbones. Interestingly, the medium-flexible connection has the highest rupture forces, whereas the ligands with highest and lowest rigidity seem to be prone to consecutive bond rupture. The presented approach allows separating bond and backbone effects in multivalent model systems.

## Introduction

In a multivalent molecular system, two partners interact with each other through two or more non-covalent equivalent interaction centers. This principle is important in biochemistry [1] and supramolecular chemistry [2], but still not fully understood on the level of individual non-covalent interactions [3]. Synthetic supramolecular systems are ideal for a quantitative analysis of multivalency on the level of single molecules, because specific ligand design can be used to study selected parameters [4–5].

The mechanical stability of a molecular system is characterized by its rupture forces under a given loading rate. Malleability describes the ability of a protein complex or bond to deform without being disrupted and is characterized by the rupture length *r*_b_ [6]. In natural environments, hydrodynamic effects may cause forces competing with biomolecular interactions, such as the leukocyte adhesion to endothelial cells [7] or the *Escherichia coli* adhesion to uroepithelium cells [8]. The latter has been well analyzed by single-molecule force spectroscopy [9], showing unfolding of a very malleable, helical PapA-domain in p-pili, which plays an important role for the adhesion. This process consumes a large amount of energy, whilst adhesion forces are kept at a low force of 27 pN, in order not to break the adhesive glycolipid–ligand interaction. In contrast, cellulosome–adhesion complexes tighten under load, leading to rupture forces of 600–750 pN, one of the strongest biomolecular interactions discovered until now [10]. Another fascinating biological example is the von Willebrand factor, where nature utilizes shear forces on an ultra large protein as self-regulative mechanism. This protein is activated by hydrodynamic forces occurring in injured blood vessels to promote hemostasis [11]. Mechanical stabilities are also of growing interest for the design of biomaterials mimicking the muscle protein titin [12–13] and smart polymers including latent catalysts for self-healing, mechanochroism or mechanoluminescence [14].

The thermal stability of a molecular system is inversely proportional to the thermal off-rate. However, this alone gives an incomplete image of bond rupture under physiological conditions. For example, depending on the direction of applied forces, the green fluorescent protein shows most-probable rupture forces between 100 pN and 550 pN at pulling speeds of 2 µm/s, but only one thermal pathway of denaturation [15]. Also the mechanical stability of the titin-telethonin complex is highly directed [16]. Instead molecular interactions in biological systems are characterized by a balanced interplay between mechanical stability and malleability. Already in 1999 Rief et al. compared the mechanical stability of the α-helical domain spectrin with refolding forces from domain I27 of the muscle protein titin in β-sheet conformation. While rupture lengths increased from 0.3 nm for titin to 1.5 nm for spectrin, the corresponding rupture forces decreased by the same ratio [17]. In 2007 it was still not clear, whether this interplay follows a linear or non-linear power law [18]. Only recently more experimental data became accessible and in 2013 Hoffmann et al. found an inverse proportional power law [6]. The rupture lengths of proteins range from 0.14 to 2 nm, while proteins with low malleability exhibit higher mechanical stability and vice versa. Then different domains of malleability were successfully attributed to mechanical clamp motifs, as suggested by Sikora et al. [19]. For example, zipper-type unfolding requires less force than shear-type denaturation. However due to the high complexity of biological multivalent interactions less is known about the influence from individual non-covalent interactions. Here, well defined model systems with known valency are valuable tools to be studied by single-molecule force spectroscopy (SMFS).

In SMFS experiments a modified probe – an optical tweezers bead or an SFM cantilever – is used to measure interaction forces with modified synthetic or biological surfaces. The distribution of interaction forces is a measure for mechanical stability. Polymeric spacers are used to detect non-specific interactions and simultaneous bond rupture. In dynamic force spectroscopy (DFS) most-probable rupture forces are measured for various pulling speeds and analyzed according to the Kramers–Bell–Evans (KBE) model, finally giving the rupture length *r*_b_ and the thermal off-rate *k*_off_ (a measure of the inverse thermal stability) [20–22]. This method is especially useful in the case of interactions with low affinity of low yield that are inaccessible for ensemble measurements. For example in 2009 Wollschläger et al. successfully detected a different binding of DNA to the corresponding domain in the transcription factor PhoB from *Escherichia coli* for the wild-type and slightly modified mutants [23]. Utilizing the sequential unzipping of transmembrane proteins, a full mechanical mapping was possible for the β_2_-adrenergic receptor [24] and rhodopsin [25]. On the field of supramolecular model systems DFS revealed the mechanical stability of coordination bonds [26–28], host–guest systems [29–32], and rotaxanes [33].

**Figure 1:**
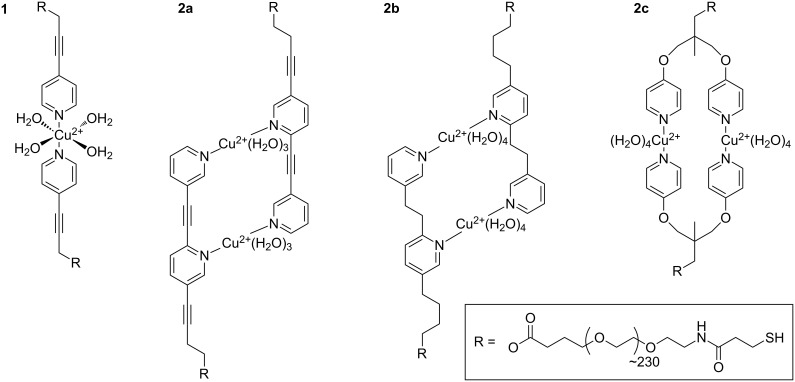
Expected coordination complexes of monovalent and bivalent structures (**1** and **2a**–**c**, respectively) with copper ions in aqueous solution. The octahedral conformation of **1** with additional water ligands was suggested by ab-initio calculations [27]. For **2a** a quasi-octahedral configuration was calculated with only three water ligands per Cu^2+^ due to steric reasons [27]. Schemes for **2b** and **2c** are suggested accordingly.

In 2008 Guzman et al. analyzed hydrogen bonds of 4H, 6H and 8H chains in toluene as model for β-sheet force clamps. They suggested that the force is transferred evenly to each hydrogen bond, giving 15 pN at a pulling speed of 200 nm/s [34]. In contrast equilibrium constants of tetravalent hydrogen bonds in chloroform are strongly dependent on arrays of donor (D) and acceptor (A) sites due to cooperative effects. Thus DAD-ADA pairs are thermally weaker than DDD-AAA complexes [35]. In 2011 Embrechts et al. showed that such cooperative effects also influences the mechanical stability of tetravalent interactions [36]. They performed DFS on UAT dimers (DADA–ADAD pairs) with UPy dimers (DDAA–AADD pairs) in hexadecane. The UPy dimers exhibit shorter rupture lengths of 0.20 nm compared to 0.29 nm, resulting in much higher rupture forces from 150 to 250 pN compared to 50–100 pN for UAT dimers. Another model system probing π–π-interactions associated with van-der-Waals forces and possible hydrophobic interactions was published in 2009 by Zhang et al. [37]. They compared the monovalent interaction of a porphyrin ligand to a C_60_ fullerene with the bivalent interaction of two ligands to one C_60_ (pincer complex) in aqueous environment. Thereby the rupture length decreased from 0.31 nm to 0.20 nm, leading to an increase in rupture forces. In contrast we recently found a model system with the opposite effect [27]. The bivalent interaction of a pyridine nanorod **2a** with Cu^2+^ showed a much longer rupture length of 0.51 nm compared to 0.33 nm for the monovalent interaction **1** in aqueous solutions of CuSO_4_ (Figure 1). As a result, rupture forces of both valencies were similar. Combining DFS with ab-initio calculations we suggested a stepwise bond-rupture including a hydrogen-bound intermediate. Thus in our system the bivalent effect did not increase the mechanical stability, but the malleability of the interaction.

In the present work we address the question, if it is possible to tune the balanced interplay between most-probable rupture forces and rupture lengths by changing the backbone connection of the pyridine model system into more flexible analogues. By performing DFS according to the KBE model we show that the rupture length may be similar to the monovalent rupture length for the system with medium flexibility **2b** (2 sp^3^ carbons in the backbone, *r*_b_ = 0.30 nm) and even larger for the system with high flexibility **2c** (3 sp^3^ carbons + 2 ether groups in the backbone, *r*_b_ = 1.12 nm). Consequently, the interaction of **2b** exhibits higher mechanical stability, but the interaction of **2c** exhibits even less mechanical stability than the monovalent interaction for the whole accessible range of pulling speeds. We will discuss possible mechanisms of simultaneous and successive bond rupture.

## Results and Discussion

**Scheme 1:**
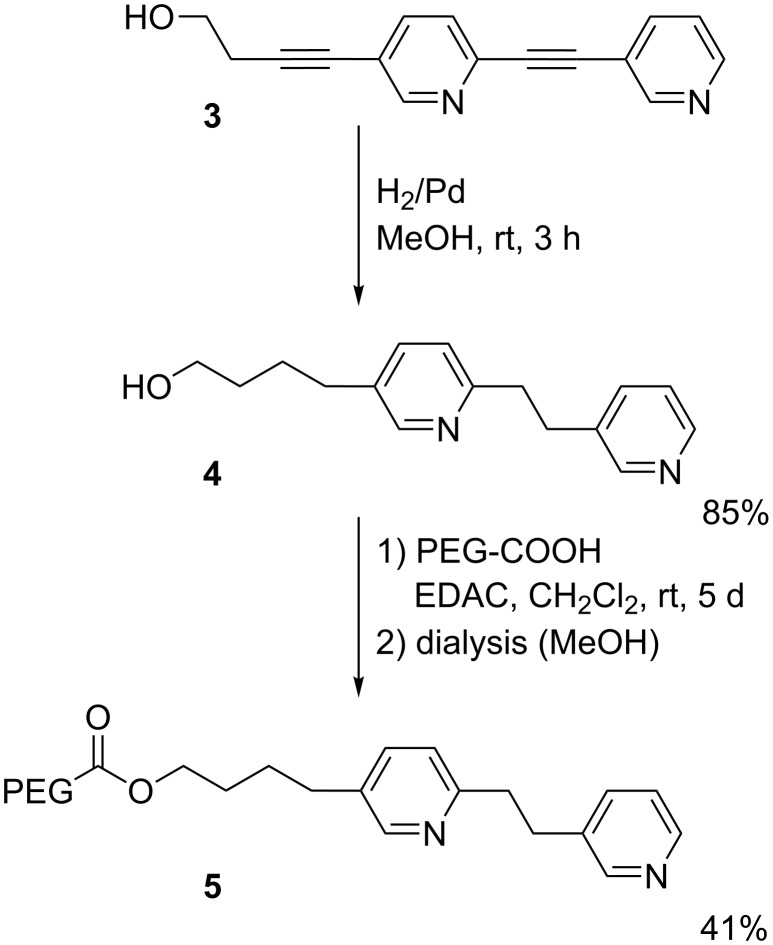
Synthesis of pyridine-PEG conjugate **5**.

**Scheme 2:**
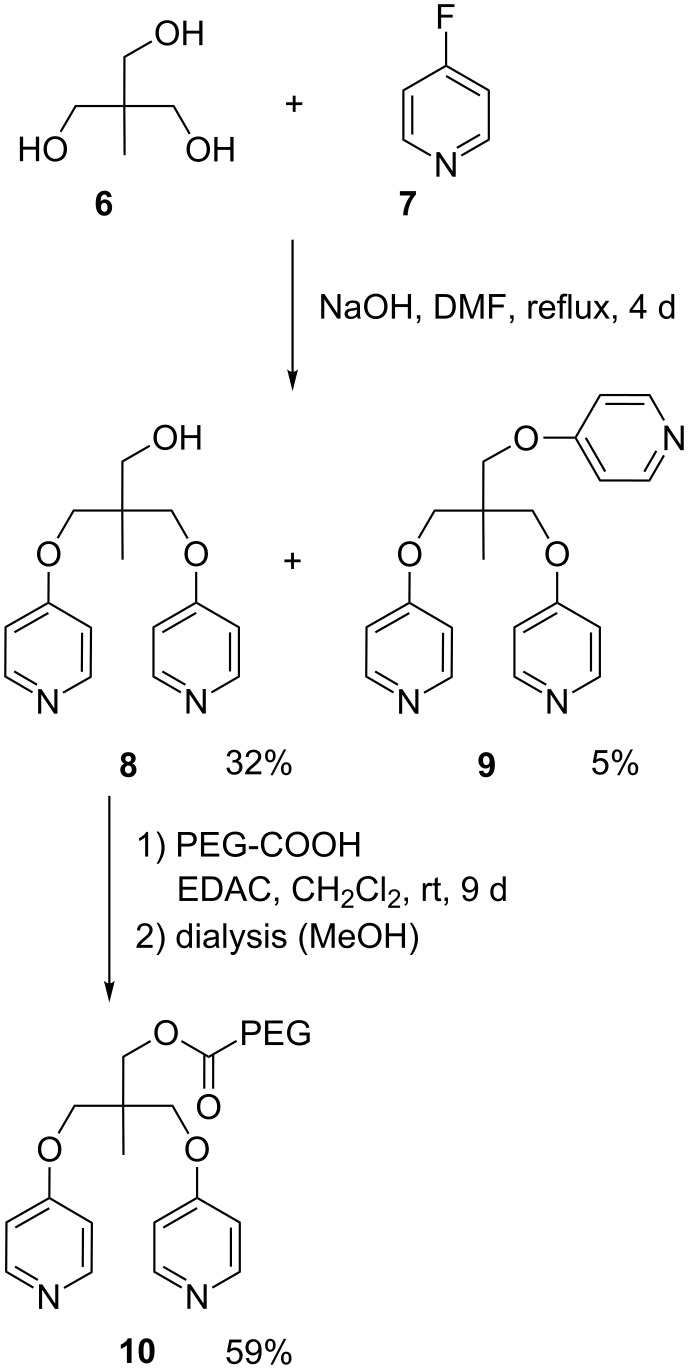
Synthesis of pyridine-PEG conjugate **10**.

Regarding the synthesis, pyridine nanorod **3**, which was also precursor for the synthesis of complex **2a** [27], was hydrogenated to receive intermediate **4** (Scheme 1). Subsequent coupling with bifunctional poly(ethylene glycol) (PEG) and purification by dialysis gave compound **5** – the ligand of coordination complex **2b**. The synthesis of compound **10** – the ligand of coordination complex **2c** – started by nucleophilic aromatic substitution of fluorinated pyridine **7** with compound **6** to receive a mixture of products **8** and **9** (Scheme 2). Purified compound **8** was then coupled with bifunctional PEG as written above.

**Figure 2:**
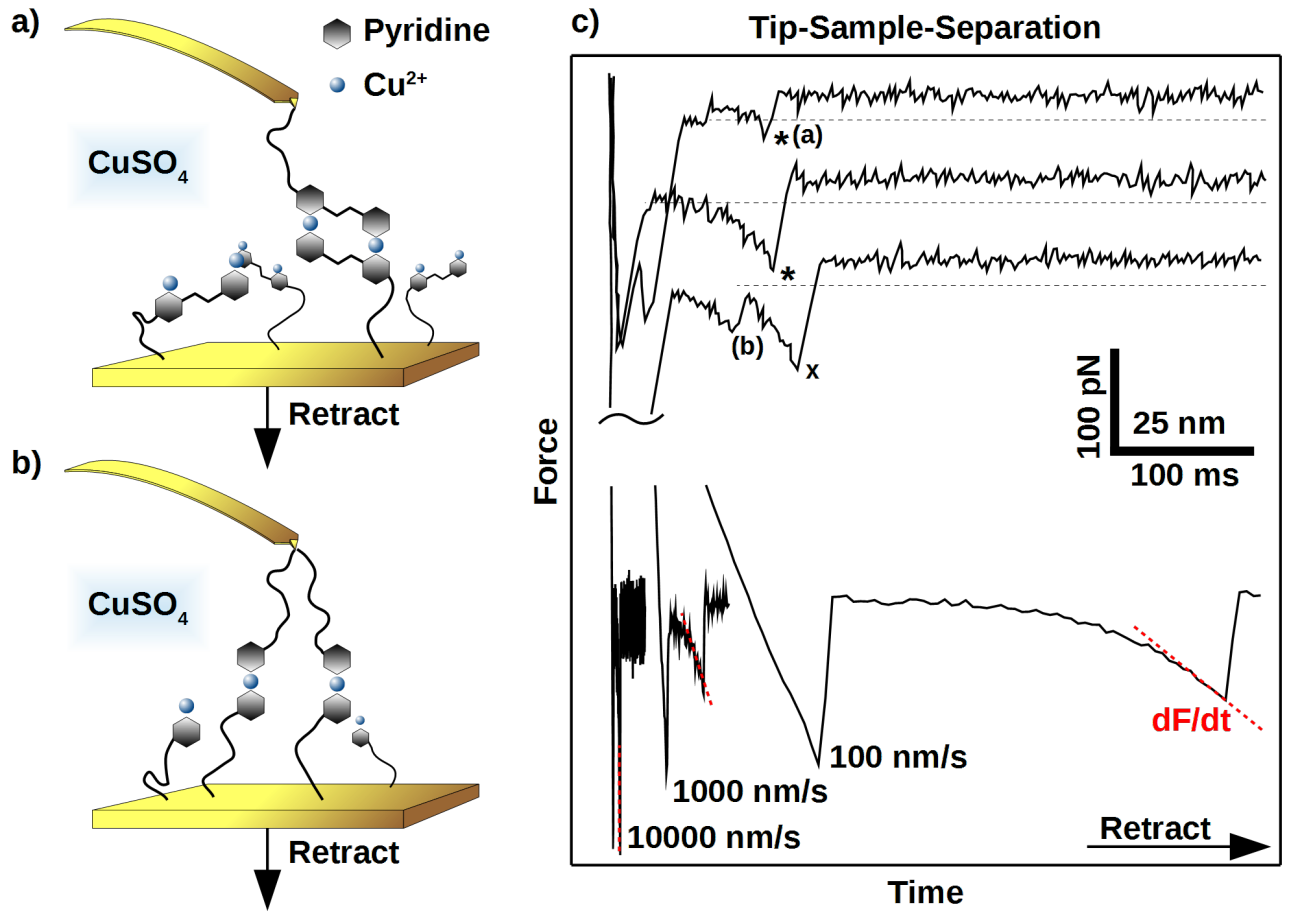
Principle of the SMFS experiment. During retraction of the sample, possible interactions are probed by bending of the calibrated SFM cantilever. a) In a single-molecule rupture event, only one mono- or bivalent ligand is responsible for the last rupture event. b) Possible simultaneous bonds, leading to multiple peaks in force–distance plots. c) Examples of force–retract behavior, plotted against tip–sample-separation (top) or experimental time frame (bottom). Signals marked with x are attributed to simultaneous bond rupture and were discarded. Dashed red lines show the loading rate dF/dt, strongly increasing from slow to fast pulling speeds (100 to 10000 nm/s). Plots are shifted for clarity.

Figure 1 shows the expected coordination complexes of our mono- and bivalent model systems with Cu^2+^ in aqueous solution. In the present work, we analyzed the bivalent systems **2b** and **2c** by DFS, similarly as for the recently published reference case of **2a** [27]. We modified gold coated SFM cantilever probes and surfaces using thiol chemistry (for details see Experimental section below). The interaction between gold and SH-groups is known to withstand rupture forces in the range of 1 to 2 nN [38], followed by the formation of a monoatomic gold nanowire that finally leads to a breakage of gold–gold bonds [39]. The rupture forces of our systems were one order of magnitude smaller, enabling repeated measurements of typically 1000 times per data point without tearing molecules off the SFM cantilever probe. The experimental setup is sketched in Figure 2a, where a bivalent complex of **2b** has already formed during a variable contact time between cantilever and sample. By retracting the sample from the cantilever, the same force is applied to the transition metal complex and the calibrated cantilever spring. Due to the finite size of a scanning force microscope (SFM) cantilever tip, there is also a certain possibility of simultaneous bond formation as shown in Figure 2b, or non-specific interactions directly between tip and surface. For a proper detection of single-molecular events, we utilized PEG chains with a well characterized force-extension behavior [40–41], resulting in a saw tooth signal at sufficiently high tip-sample-separation (Figure 2c, top). Of each force-distance experiment, only the last peak was selected, if it showed the characteristics of a PEG chain and a starting value lower than 6 times the root-mean-square (rms) thermal background noise (dashed line). The latter is important to ensure a proper application of the KBE model. For DFS, measurements have to be performed over a broad range of pulling speeds, resulting in various loading rates (dF/dt) as illustrated in the bottom of Figure 2c.

A simultaneous bond rupture of the system as sketched in Figure 2b would be that of a bivalent system as well. However in contrast to bipyridines **2a**–**2c**, the entropic elasticity of the spring would instantaneously pull away the first bond that is broken, making rebinding effects impossible [42]. Thus rupture forces in such cases are additive [43].

**Figure 3:**
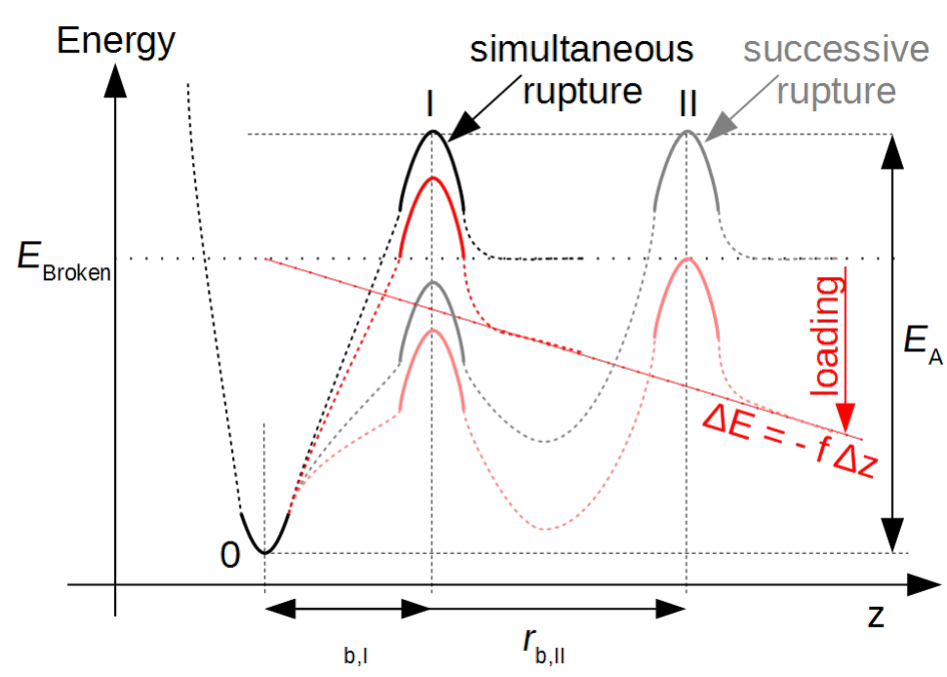
Potential energy diagrams according to the KBE model for simultaneous and successive bond rupture are only characterized by a bound state 0, and one dominant sharp transition state I or II. Loading of a bond deforms the potential energy along the rupture coordinate z according to ∆*E*, thereby reducing activation energy *E*_A_ and increasing force-driven rupture *k*_off_(f).

In this study we aimed at detailed information on the rupture behavior of the model systems described above. We utilized the frequently employed KBE model [20–22] to calculate rupture length *r*_b_ (a measure of malleability) and *k*_off_ (a measure of the inverse thermal stability). The model makes some assumptions to a hypothetical potential energy diagram (PED) along the rupture coordinate z (Figure 3). Starting from a bound state 0, a certain activation energy *E*_A_ is needed to escape over transition state I. Under an applied force, *f*, the whole PED is tilted by ∆*E* = −*f* ∆*z*, where ∆*z* is the distance from state 0. As a consequence the potential wall of the transition state I is lowered by ∆*E* = − *f r*_b,I_ and the probability of bond rupture is increased. In a SMFS experiment, the force is increased by a certain loading rate, proportional to the pulling speed, and faster loading rates lead to higher average rupture forces. Systems with successive bond rupture have a second transition state II with higher rupture length *r*_b,II_. If II is of higher energy than I, it is the dominant transition state and rupture is much more sensitive to forces.

Under the assumption of a constant loading rate, the KBE model can be solved analytically resulting in Equation 1, where *k*_B_ is the Boltzmann constant and *T* the absolute temperature. A good derivation of Equation 1 and comparison with non-constant loading rate can be found in [44]. Note that the value of *k*_off_ describes the thermal off-rate along the mechanical reaction coordinate. Especially in complex systems, other dissociation paths with different thermal off-rate are possible.

[1]
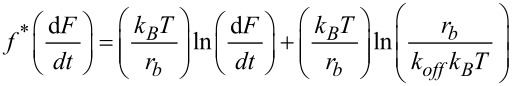


**Figure 4:**
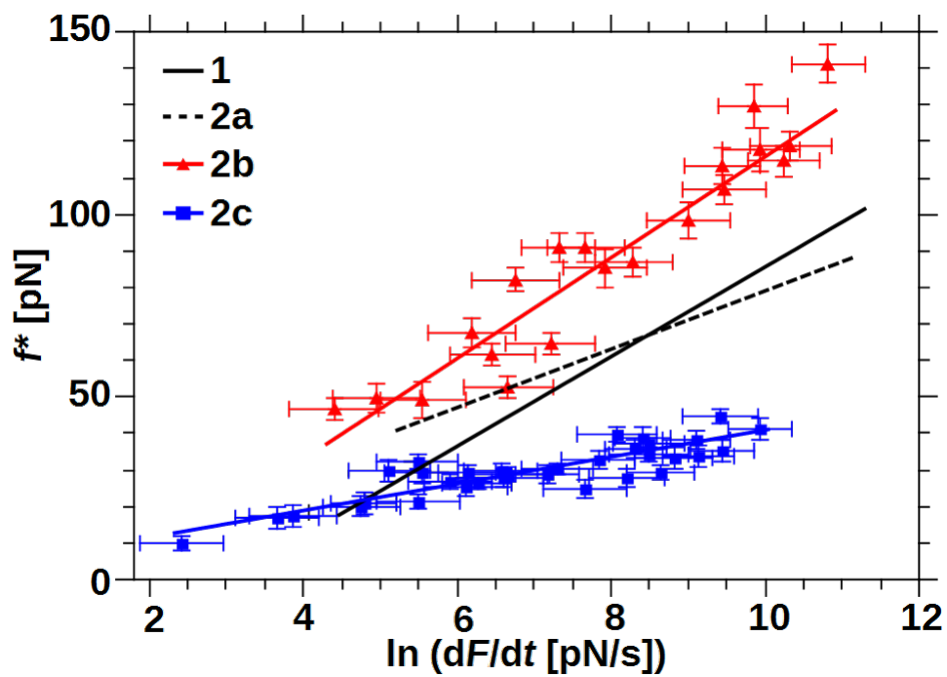
Most probable rupture forces plotted over their corresponding loading rate. Each point denotes for one series of measurements at a certain pulling speed. Error bars: f* – standard error of rupture force histogram, ln(d*F*/d*t*) – average loading rate error of all rupture events in the range of f*. KBE fits are shown according to Equation 1, black lines according to [27].

If experimental results of a DFS experiment show a linear behavior of most probable rupture forces f* with ln(d*F*/d*t*) (Figure 4), the KBE model may be applied. Then the slope is a measure of the inverse rupture length *r*_b_ and the x-intercept a measure of *k*_off_. Two linear regimes denote a change in two dominant transition states [45], a non-linear behavior may be due to a more complex PED [46]. A drawback of this method is the reduction of all measured rupture forces to one most-probable force value. Some groups expanded the KBE model to directly fit the whole data set, taking bond heterogeneity [47] or a temperature dependent Arrhenius prefactor into account [48–49]. We thoroughly applied the first mentioned model to our results as well, but did not obtain consistent results. This may be partially due to the fact that the π–π-stacking of pyridines [50] was a competing interaction. The most probable rupture force, used in the KBE model, was due to the coordination complexes. Methods using the whole data set are strongly influenced by the stacking interaction and would have needed heavily time consuming adaption for a proper fit of our data. This was beyond the scope of this work.

Both bivalent systems analyzed in this study could be described by the linear KBE model fit (Figure 4, Table 1). System **2b** exhibited the highest rupture forces over the whole range of measured loading rates. The slope was similar to the monovalent interaction of **1**, resulting in similar rupture lengths. In contrast, rupture forces of system **2c** were smaller than the values for **2a** and **2b** and even lower compared to the monovalent interaction of **1** at loading rates larger than ln(d*F*/d*t*) = 5. The flat slope of system **2c** resulted in the largest rupture length of all systems analyzed. System **2a** analyzed previously had a medium-slope and crossed the x-axis at a similar loading rate to system **2c**, giving similar thermal off-rates.

**Table 1:** KBE model fit results for systems **2b** and **2c**, analyzed in this work, compared with values for **1** and **2a** from [27].

	*r*_b_ [nm]	*k*_off_ [s^−1^]

**1** [27]	0.33 ± 0.01	1.7 ± 0.2
**2a** [27]	0.51 ± 0.03	0.14 ± 0.06
**2b**	0.30 ± 0.01	0.36 ± 0.07
**2c**	1.12 ± 0.07	0.09 ± 0.04

The rupture lengths of systems **1** and **2a** are surprisingly high for interactions on the single-molecular level. For example, a Pd^2+^ pincer complex with two different pyridine ligands shows rupture lengths around 0.2 nm in DMSO [28]. Using ab-initio calculations we could show that a hydrogen-bound intermediate state stabilizes the interaction over a longer distance [27]. In this case, a water molecule from the solvent jumps into the pyridine–Cu^2+^ interaction. Thus we assume a similar dissociation process for **2b** and **2c**. In DMSO such an effect is not possible and the single transition state around 0.2 nm is rate determining. Unfortunately CuSO_4_ is insoluble in DMSO, preventing corresponding experiments on our systems.

The medium-flexible bipyridine **2b** interacts with a similar rupture length as the monovalent system, but the thermal off-rate is significantly smaller and comparable to system **2a**. Therefore a different supramolecular complex with higher mechanical stability and lower thermal off-rate has been formed. We propose a bivalent interaction similar to the known complex **2a** as shown in Figure 1. From a PED point of view, this behavior is described either by a simultaneous bond rupture as shown in Figure 3, I, or by a successive rupture process (Figure 3, II) where the first transition state is rate dominating. However the KBE model fit for the stepwise process would only extrapolate to the thermal off-rate of the first transition state, gaining the value for a monovalent interaction. Thus in the following discussions we will suggest a simultaneous rupture.

**Figure 5:**
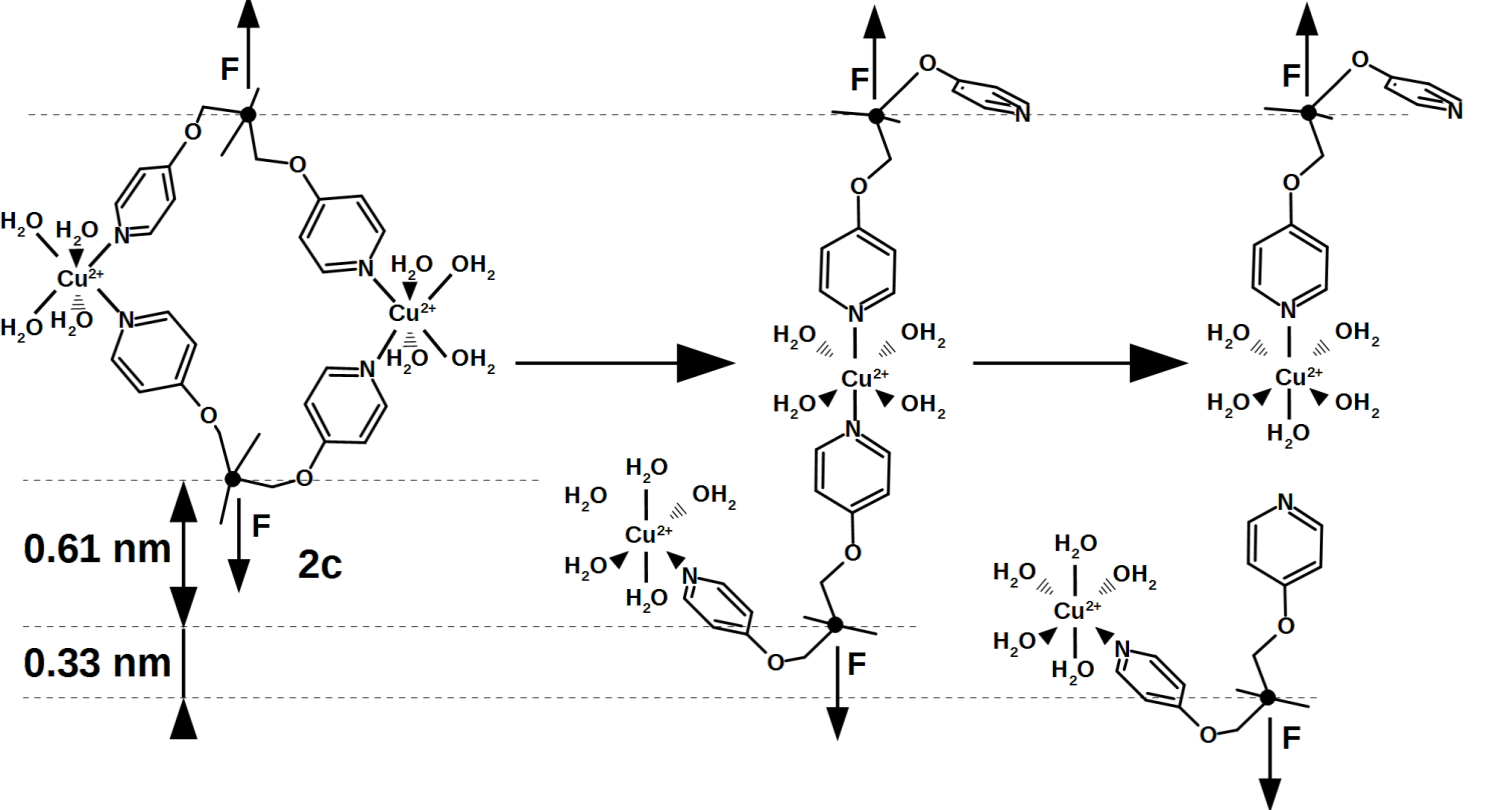
Possible rupture mechanism describing the extraordinary long rupture length of system **2c**. Starting from an ortho-configuration, the first bond rupture leads to an increased distance between both force-points (emphasized by force vector arrows). The second jump is attributed to a hydrogen-bound intermediate found in the monovalent rupture process. Structures were calculated using the molecular mechanics tool based on CHARMM force fields included in ChemSketch (v 14.01, ACD/Labs, Toronto, Canada).

The lower thermal off-rate of the flexible pyridine **2c** compared to **1** also indicates a bivalent interaction. In contrast to **2b**, the rupture length is much larger and we suggest a stepwise bond rupture such as PED II in Figure 3. Still the rupture length of 1.12 nm is too large to be described by the known hydrogen-bound intermediate alone. A possible explanation is the release of geometrical folding after breakage of the first bond. A purely geometrical molecular mechanics estimation of a possible ortho-complex of both Cu^2+^ metal centers results in a 0.61 nm length increase after rupture of the first bond due to the applied strain (Figure 5). The maximum velocity in the DFS experiment is very slow on the atomic length scale, thus the remaining complex could also switch to para-configuration. After additional 0.33 nm rupture length for a monovalent interaction, an overall rupture length around 0.94 nm would be gained by the KBE model. This value is already close to the experimentally observed length.

**Figure 6:**
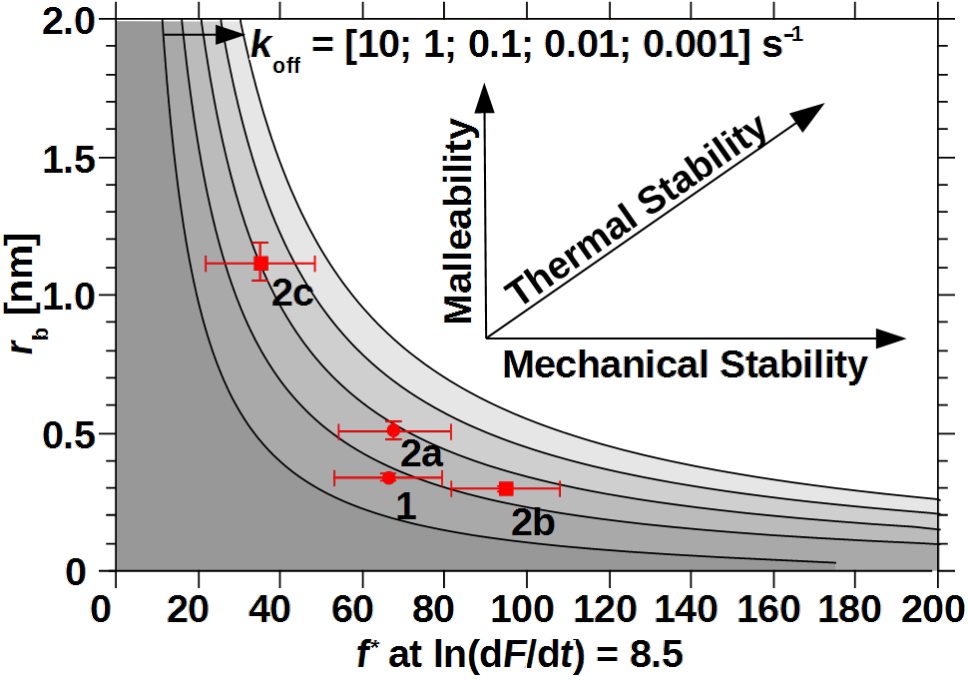
Most probable rupture forces at a logarithmic loading rate of 8.5 in relation to the corresponding rupture lengths of monovalent and all three bivalent interactions. Continuous lines denote for numerically calculated rupture lengths, according to the KBE model (Equation 1) for exponentially decreasing thermal off-rates. Circles: data from [27]; squares: this work; x-error bars: average standard error of measurements around the given loading rate, y-error bars: uncertainty according to KBE fit.

The mechanical stability, namely the most probable rupture force f*, of a system that follows the KBE model strongly depends on the applied loading rate. Two interactions with different rupture length may have a crossing of their force-loading-rate behavior. If the intersection is outside the experimental accessible area, a ranking of mechanical stability can still lead to a deeper understanding of the rupture behavior and will be discussed in the following [6,18]. We have chosen a medium loading rate of ln(d*F*/d*t*) = 8.5 that is just at the crossing of **1** and **2a**, emphasizing their similar forces over the whole range of experimental loading rates. Also **2b** has the largest and **2c** the smallest rupture forces of all results at this loading rate, reflecting their overall behavior. Figure 6 shows the most probable rupture forces at this loading rate in relationship to their rupture length r_b_. The plot also includes the expected balance between *r*_b_ (malleability) and f* (mechanical stability) over five orders of magnitude in thermal off-rate, according to the KBE model Equation 1. Especially in the high-force and high malleable regimes those “isoenergetic” lines are close by, due to their logarithmic influence. Thus an interaction would need a very small thermal off-rate to combine, for example, malleability and mechanical stability. On the other hand a mechanically very stable system with average thermal off-rate may be gained by a reduction of the rupture length. Such an example was recently published for the cellulosome-adhesion complex, where the force-shielding subdomain XMod drops the rupture length from 0.19 nm to 0.13 nm and decreases the thermal off-rate by three orders of magnitude to finally raise the rupture forces from 280 pN to 610 pN at ln(d*F*/d*t*) = 8.5 [10]. On the single-molecular level, the interaction between Zn-porphyrine and C_60_ fullerenes shows a similar trend comparing their monovalent and bivalent interaction [37]. The latter has a shorter rupture length, lower thermal off-rate and consequently higher most probable rupture forces. In contrast our measurements on three bivalent model systems with similar coordination complexes and thus similar thermal off-rates showed a new possible trend for system **2c**. Here, the rupture length increase overcompensated the gain in mechanical stability, leading to even lower rupture forces for loading rates down to ln(d*F*/d*t*) = 5 (see also Figure 4). System **2b** with medium flexibility showed the trend, already known for other systems [30].

## Conclusion

The rupture behavior of three bivalent molecular model systems was varied over a broad range of rupture lengths and most probable rupture forces, employing backbones with different geometries and flexibilities. While the interactions lead to similar thermal off-rates, the rupture mechanisms are different. The system with medium backbone flexibility shows a simultaneous bond rupture, leading to a high mechanical stability. On the other hand, a stepwise rupture processes, possibly combined with an additional release of geometrical folding, results in a very malleable system that is able to deform without breaking. Thus knowledge about backbone properties of bivalent and probably also multivalent interactions is crucial for the specific design of ligands. Future studies will address specific backbone properties and higher valencies on the way to a deeper understanding of their influence on multivalency.

## Experimental

Reactions were generally performed under argon in dried flasks. Solvents and reagents were added by syringes. Solvents were dried using standard procedures. Dichloromethane was dried with activated alumina using an MBraun solvent system model MB SPS-800. Other reagents were purchased and used as received without further purification unless otherwise stated. Reactions were monitored by thin-layer chromatography (TLC). Products were purified by flash chromatography on silica gel (32–63 μm, Macherey & Nagel). Yields refer to chromatographically and spectroscopically (^1^H NMR) homogeneous materials, unless otherwise stated. NMR spectra were recorded on Bruker (AM 250, AC500, AVIII 700) and JEOL (ECX 400, Eclipse 500) instruments. Integrals are in accordance with assignments, and coupling constants are given in Hz. Chemical shifts are reported relative to TMS (^1^H: δ = 0.00 ppm) and CDCl_3_ (^13^C: δ = 77.0 ppm). All ^13^C NMR spectra are proton decoupled. For detailed peak-assignment 2D spectra were measured (COSY, HMQC, HMBC). Multiplicity is indicated as follows: s (singlet), d (doublet), t (triplet), m (multiplet), m_c_ (centered multiplet), dd (doublet of doublet), br s (broad singlet). IR spectra were measured with a Nexus FTIR spectrometer equipped with a Nicolet Smart DuraSampleIR ATR. MS and HRMS analyses were performed with Varian Ionspec QFT-7 (ESI–FT ICRMS) instrument. Elemental analyses were carried out with a Vario EL III analyser. Melting points were measured with a Reichert Thermovar apparatus and are uncorrected.

**Synthesis of 4-{6-[2-(pyridin-3-yl)ethyl]pyridin-3-yl}butan-1-ol (4)**: A suspension of 4-{[2-(pyridin-3-yl)ethynyl]pyridin-5-yl}but-3-yn-1-ol (**3**) [27] (45 mg, 0.18 mmol) and Pd/C (45 mg, 100 wt %) in MeOH (3 mL) was stirred under an atmosphere of hydrogen (balloon) for 3 h until complete consumption of the starting material (by TLC). The mixture was filtered through a short plug of silica gel (MeOH) and evaporated to afford 39 mg (85%) of product **4** as a colorless oil. ^1^H NMR (400 MHz, CDCl_3_) δ 1.16–1.55, 1.66–1.73 (2 m, 2H each, 2-H/3-H), 2.62 (t, *J* = 7.5 Hz, 2H, 4-H), 3.05 (s, 4H, 1’-H/2’-H), 3.64 (t, *J* = 6.4 Hz, 2H, 1-H), 6.94 (d, *J* = 7.9 Hz, 1H, pyr), 7.19 (dd, *J* = 4.8 Hz, 7.9 Hz, 1H, pyr), 7.38 (dd, *J* = 2.3, 7.9 Hz, 1H, pyr), 7.46–7.61 (m, 1 H, pyr), 8.31 (br s, 1 H, pyr), 8.37–8.41 (m, 2 H, pyr) ppm; ^13^C NMR (101 MHz, CDCl_3_) δ 27.3, 32.1, 32.3, 33.0 (4 t, C-2/C-3/C-1’/C-2’), 39.0, 62.2 (2 t, C-4/C-1), 122.7, 123.3 (2 s, pyr), 135.2, 136.0, 136.3, 136.8, 147.2, 149.3, 149.7 (7 d, pyr), 157.6 (s, pyr) ppm; IR (ATR) ν: 3305 (OH), 3030–2860 (=C-H, -C-H), 1600–1570 (C=C) cm^−1^; HRMS (ESI–TOF): *m/z* [M + H]^+^ calcd for C_16_H_21_N_2_O, 257.1648, found, 257.1634.

**Synthesis of pyridine-PEG conjugate 5, ligand of 2b:** To a solution of the bifunctional poly(ethylene glycol) (R in Figure 1, H-terminated, 81.0 mg, 0.0081 mmol) and **4** (29.0 mg, 0.113 mmol) in anhydrous CH_2_Cl_2_ (0.6 mL) was added EDAC (6.5 mg, 0.034 mmol) and the resulting mixture was stirred at room temperature under an atmosphere of argon for 5 days. The solvent was evaporated and the residue was purified by dialysis (MW cut-off: 1000 g/mol) against MeOH to provide the pyridine-PEG conjugate **5** (33 mg, 41%) as a colorless solid. Mp 127 °C; ^1^H NMR (700 MHz, CDCl_3_) δ 1.54–1.62, 1.64–1.72 (2 m, 2H each, C-2/C-3), 2.62 (m, 2H, 4-H), 3.06 (s, 4H, 1’-H/2’-H), 3.64 (s, OCH_2_CH_2_O), 6.94 (t, *J* = 8.0 Hz, 1H, pyr), 7.19 (dd, *J* = 5.3, 7.5 Hz, 1 H, pyr), 7.34–7.41 (m, 1 H, pyr), 7.50 (d, *J* = 7.7 Hz, 1 H, pyr), 8.30 (br s, 1 H, pyr), 8.38–8.44 (m, 2 H, pyr) ppm.

**Synthesis of 2-methyl-3-(pyridin-4-yloxy)-2-[(pyridin-4-yloxy)methyl]propan-1-ol (8) and 1,1,1-tris[pyridine-4-yloxy)methyl]ethane (9):** To a solution of 2-(hydroxymethyl)-2-methylpropane-1,3-diol (313 mg, 2.33 mmol) in dry DMF (25 mL) was added NaOH (600 mg, 15.0 mmol). After 15 min stirring at room temperature, 4-fluoropyridine (777 mg, 8.00 mmol) was added. The mixture was heated to reflux for 4 days and after cooling to room temperature diluted with CH_2_Cl_2_/water. The organic phase was washed several times with water and then dried (Na_2_SO_4_). Column chromatography on aluminum oxide (CH_2_Cl_2_/MeOH 94:6) afforded 338 mg of a yellowish solid (mixture of **8** and **9**) and 21 mg (3%) of compound **8** as colorless solid. By further purification steps (second chromatography on silica gel followed by HPLC) additional **8** (188 mg, 29%) and **9** (44 mg, 5%) were isolated.

**Data of compound 8:** Mp 161–162 °C; ^1^H NMR (400 MHz, CD_3_OD) δ 1.19 (s, 3H, Me), 3.68 (s, 2H, 1´-H), 4.09 (m_c_, 4H, OCH_2_), 7.00 (d, *J* = 4.5 Hz, 4H, 3-H, 5-H), 8.33 (m_c_, 4H, 2-H, 6-H) ppm; ^13^C NMR (101 MHz, CD_3_OD) δ 17.1 (q, Me), 42.0 (s, C-2´), 64.7 (t, OCH_2_), 71.0 (t, OCH_2_), 111.9 (d, C-3, C-5), 155.7 (d, C-2, C-6), 159.7 (s, C-4) ppm; IR (ATR) ν: 3135 (OH), 3100–3025 (=C-H), 2960–2865 (C-H), 1590–1460 (C=C, C=N), 1055–1025 (C-O) cm^−1^; HRMS (pos. ESI-TOF) *m/z*: [M + H]^+^ calcd for C_15_H_19_N_2_O_3_, 275.1396; found, 275.1403; anal. calcd for C_15_H_18_N_2_O_3_: C, 65.68; H, 6.61; N, 10.21; found: C, 65.17; H, 6.45; N, 10.13.

**Data of compound 9:** Mp 143–144 °C; ^1^H NMR (250 MHz, CDCl_3_) δ 1.33 (s, 3H, Me), 4.10 (s, 6H, OCH_2_), 6.80 (m_c_, 6H, 3-H, 5-H), 8.41 (m_c_, 6H, 2-H, 6-H) ppm; ^13^C NMR (63 MHz, CDCl_3_) δ 17.3 (q, Me), 40.2 (s, C-1´), 69.4 (t, OCH_2_), 110.3 (d, C-3, C-5), 151.3 (d, C-2, C-6), 164.6 (s, C-4) ppm; IR (ATR) ν: 3050–3035 (=C-H), 2950–2870 (C-H), 1685–1455 (C=C, C=N), 1110 (C-O) cm^−1^; HRMS (pos. ESI–TOF) *m/z*: [M + H]^+^ calcd for C_20_H_22_N_3_O_3_, 352.1656; found, 352.1659; anal. calcd for C_20_H_21_N_3_O_3_: C, 68.36; H, 6.02; N, 11.96; found: C, 68.02; H, 6.00; N, 11.93.

**Synthesis of pyridine-PEG conjugate 10, ligand of 2c:** To a solution of the bifunctional poly(ethylene glycol) (R in Figure 1, H-terminated, 117 mg, 0.0117 mmol) and compound **8** (32.0 mg, 0.116 mmol) in anhydrous CH_2_Cl_2_ (0.6 mL) was added EDAC (7 mg, 0.035 mmol) and the resulting mixture was stirred at room temperature under an atmosphere of argon for 9 days. The solvent was evaporated and the residue was purified by dialysis (MW cut-off: 1000 g/mol) against MeOH to provide the pyridine-PEG conjugate **10** (69 mg, 59%) as a colorless solid (mp. 55–58 °C). **^1^**H NMR (CDCl_3_, 700 MHz) δ 6.81 (dd, *J* = 4.8, 1.5 Hz, 4H, 3-H, 5-H), 8.43 (dd, *J* = 4.8, 1.5 Hz, 4H, 2-H, 6-H) ppm.

Surface films of the polymers were prepared in a similar manner as described before [27]. A droplet of a 1 mM aqueous polymer solution was applied to freshly template-stripped gold supports [51] (JPK Instruments, Berlin, Germany) for 12−24 h at room temperature in an enclosed chamber with water reservoir. Immediately before measurements the surfaces were thoroughly rinsed with water [52]. For blank experiments, surfaces were stripped and used without further treatment. Gold-coated Si_3_N_4_ cantilevers (Olympus Biolever, 60 µm short cantilever: *k* = 0.03 N/M, 100 µm long cantilever: *k* = 0.006 N/m, Olympus Corp., Tokyo, Japan) were cleaned by UV/O_3_ treatment [53] (Penray low-pressure mercury discharge tube, UVP, Upland, CA) and treated as gold supports above.

SMF measurements were performed as in [27], i.e., on a ForceRobot 200 [54] (JPK Instruments, Berlin, Germany) at room temperature in an enclosed fluid cell filled with 3 mM CuSO_4_ (**2b**), 30 mM CuSO_4_ (**3c**) or DI water (blank). SFM cantilevers were calibrated using the thermal noise method [55]. In DFS, experiments were performed at constant velocities between 100 nm/s and 10 µm/s using a grid of different spots on the surface.

Force–distance curves were processed as described in [27]. In short, signals were fitted according to the wormlike-chain model using Hooke, a Python-based force spectroscopy data analysis program [56]. Most probable rupture forces were determined by histogram analysis. Loading rates at the rupture point of each curve were calculated based on the fit function and pulling velocity. Measurements in aqueous solutions without CuSO_4_ (blank) showed a different force-loading rate behavior, proving specific interactions with the Cu^2+^ ligand. In 3 mM CuSO_4_, ligand **2c** showed the same behavior as the monovalent system **1**, but a different in 30 mM CuSO_4_. The latter was used for the analysis presented here.
